# Vision System for Coarsely Estimating Motion Parameters for Unknown Fast Moving Objects in Space

**DOI:** 10.3390/s17122820

**Published:** 2017-12-05

**Authors:** Min Chen, Koichi Hashimoto

**Affiliations:** Graduate School of Information Sciences, Tohoku University, Aramaki Aza Aoba 6-6-01, Aoba-Ku, Sendai 980-8579, Japan; koichi@m.tohoku.ac.jp

**Keywords:** feature point detection, 3D reconstruction, motion estimation, camera system, robust estimation, video tracking

## Abstract

Motivated by biological interests in analyzing navigation behaviors of flying animals, we attempt to build a system measuring their motion states. To do this, in this paper, we build a vision system to detect unknown fast moving objects within a given space, calculating their motion parameters represented by positions and poses. We proposed a novel method to detect reliable interest points from images of moving objects, which can be hardly detected by general purpose interest point detectors. 3D points reconstructed using these interest points are then grouped and maintained for detected objects, according to a careful schedule, considering appearance and perspective changes. In the estimation step, a method is introduced to adapt the robust estimation procedure used for dense point set to the case for sparse set, reducing the potential risk of greatly biased estimation. Experiments are conducted against real scenes, showing the capability of the system of detecting multiple unknown moving objects and estimating their positions and poses.

## 1. Introduction

This work is considered as a significant building block in a system for analysis of flying animals, motivated by biological interests in animals’ navigation behavior. The information on the path of a target animal and its rough poses need to be measured. In this paper, our goal is to detect unknown fast moving objects within a given space, and calculate their motion parameters represented by positions and poses, using two calibrated cameras. Recognition of detected objects, i.e., whether that object belongs to a targeted category of interest, is not concerned, and will not be discussed in the contents of this paper.

We use two calibrated cameras to simultaneously capture images from different perspectives of a scene. Any object appearing in the scene will then be detected and tracked. A simple structure represented by 3D points of the detected object is reconstructed from images. This structure is then used to estimate the motions and is updated until the object disappears from scene. Here, the assumed situation is that the target might be moving fast inside the image plane, making it difficult to reconstruct points for the target. The absolute value of speed in space is not important; for example, an airplane far away in the sky, however fast it actually is, is considered slow, when viewed from a camera located on the ground.

To the best of our knowledge, this problem is not well studied. Although it can be theoretically modeled as a Structure from Motion (SfM) [1] problem, where the 3D structure of a scene needs to be reconstructed using a set of images taken from different perspectives, it differs from a typical SfM problem in several aspects: (1) In an SfM, the target is usually static, or with very slow motion, whereas, in our case, targets are moving fast, so images of good quality are not available due to motion blur; (2) The target in an SfM takes a large part of the whole scene, so almost all the information in an image is helpful, while, in our case, one target usually only takes a small portion inside an image, so available information is less; (3) SfM tries to reconstruct one single target from images, but, in our case, it is possible for multiple objects to co-exist in the scene.

In our system, we also need to reconstruct the 3D coordinates for points on target objects. However, since our goal is not to visualize a whole scene, it is not necessary to densely reconstruct as many points as is done in a typical scene reconstruction. This is because: (1) we are not interested in the background, thus background points shall be ignored; and (2) not all points on the target surface are necessary because a 6-DOF PnP problem can be solved theoretically using no more than four point correspondences in general positions, given that the four points are confident. It is desired that smaller amount of points is sufficient to carry out good estimation result.

To tackle the difficulties in reconstructing points on fast moving objects, in our system, we first proposed a novel method to detect reliable interest points on moving objects from images. With these 2D image points, point correspondences are calculated, and their 3D coordinates are reconstructed. These reconstructed points will then be selected and grouped to represent structures of moving objects. The point set for each object is maintained and updated according to a schedule taking into account changes in appearances and perspectives. Because the maintained object points is very sparse, and their images are generally not uniformly distributed inside captured frames, a conventional estimation step for dense point set will lead to biased estimation results. We adapt the robust estimation algorithm to sparse non-uniformly distributed point set for our system, and use this to find more reliable estimations.

The contents of this paper is organized as follows: Section 2 reviews studies that are related to this work. In Section 3, we introduce the main data processing work flow used in our system and some technical details on the steps in the flow. Section 4 shows the data collected from examples used in our experiments. Section 5 discusses the experiment results and the limitations of our system. Section 6 briefly summarizes the work in this paper.

## 2. Related Work

Multiple Object Tracking (MOT) is one of the most important problems in computer vision. It covers a wide range of specific tasks, with various assumptions and requirements. Although different kinds of approaches have been proposed to tackle this problem, no general solution exists.

One important factor in MOT is the assumed target. According to the specific application, the target can be pedestrians on the street [2,3], vehicles on the road [4], bats in the sky [5,6], fish under water [7], etc. Methods in these works are typically based on the prior knowledge of the specific class of assumed targets. This knowledge can be either explicitly specified by human engineering [3,4,7], or obtained though learning process [6]. The limitation is within the capability of generalization, i.e., target specific knowledge can not be easily adapted to different targets. Thus, changing the target usually means a completely new task, requiring large amount of effort, such as feature engineering or creating learning dataset.

The required information of a MOT task also varies. The majority of works in earlier studies focus on locating targets in 2D image plane [4,5,7]. Some task may also require extra information such as the approximate region, which is typical when the target is comparatively large [3]. In more recent studies, 3D trajectories of targets are reconstructed using multiple images [8]. Mostly, data association techniques are adopted to find correspondences between data samples across time sequences and multiple images [5,8,9]. However, in these works, targets are often modeled as simple points, so more advanced information is difficult to obtain.

The main problem concerned in this paper is to detect unknown fast moving objects within a given space, and coarsely estimate their positions as well as their poses, using sequence of image pairs. This task is challenging because not much prior knowledge is provided about the targets, except for a few basic assumptions. Approximate structures of targets must be reconstructed from images, and they will be used in motion estimation. As mentioned in Section 1, this problem is similar to an SfM problem, but differs in several aspects. Simply applying conventional methods used in SfM is not appropriate.

It is worth noting that this problem is different from those in [10,11,12] estimating object poses. For static scenes or where only slow motion is assumed, depth sensors such as kinect [13] can be used as powerful tools to directly get densely reconstructed point clouds, as is the case in [11,12]. It is not currently possible to directly use these sensors to obtain points of good quality on fast moving objects. In addition, models of the targets in [10,11,12] are provided from the beginning so concrete prior information, such as image textures associated with specific points, or spatial structures of objects, are available and can be used in estimation.

In our method, we also need to reconstruct the 3D coordinates for points on target objects. To do this, it is essential that point correspondences must be calculated. General approaches of finding point correspondence across multiple views can be mainly classified into two groups: direct methods [14] and feature based methods [15]. Direct methods, under the assumption of brightness constraint, recover all unknown parameters directly from measurable image quantities at each pixel, often equipped with the tool of image hierarchies. It is more suited for reconstruction of scene where the depth of the scene is relatively small compared with its distances from camera centers, in which case transformation between images can be modeled and computed using one homography while still achieving good approximation. Feature based methods, on the other hand, first detect a set of interest points, and then compute their feature descriptors. Those detected interest points will later be matched and fitted to some transformation model using robust estimation algorithms such as [16,17], before a dense correspondence map is generated for all points with the guidance of interest points correspondences.

Simply applying typical algorithms of both methods does not produce satisfactory results. For the direct methods, brightness constraint is the most fundamental assumption, and it requires that pixel values do not vary greatly across different images [14]. However, for fast moving objects, motion blur, although alleviated by adopting high speed cameras in our system, is a severe problem. The brightness of a pixel on an object is regularly varying in an unpredictable way. Besides, the depth of an object cannot be ignored because the depth information will surely affect the estimation result of poses, thus it is not appropriate to model transformations across images simply using global homographies. Consequently, point correspondences found by direct methods are not reliable.

Since features can be designed to be invariant to small changes, we consider feature based methods to be more promising in our case. However, general purpose local features such as [18,19,20,21,22] is not robust for points on moving objects. One of the reasons is that the detector algorithm for finding interest points is not well suited for points in blurred area. They are most likely to find points only in the background, since the textures there usually look far more distinct compared with those on moving objects. Besides, a typical feature calculating algorithm, whether gradient based or other, does not distinguish between foreground pixels and background pixels within the input image patch. Thus, an image patch surrounding a point near the boundary of a moving object is very likely to have varying feature value because it contains background pixels which is regularly changing. Finally, features calculated by those algorithms may also suffer from motion blur and perspective changes, although not as much compared with brightness used in direct methods.

## 3. Methods

**Figure 1:**
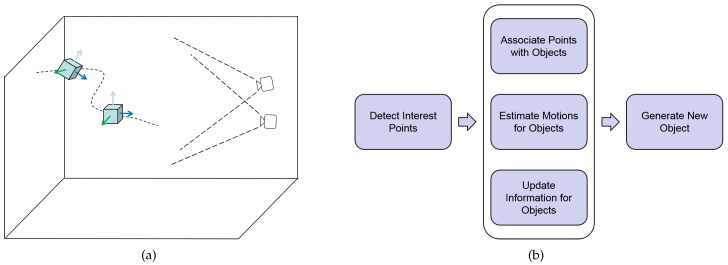
System overview. (**a**)The assumed situation for the system: The cameras are pointing at a given space where targets are expected to appear. More than one, but a few objects may co-exist in the scene. (**b**) The conceptual work flow of data processing on a single pair of image frames.

We use two calibrated cameras pointed at the same space to simultaneously capture image frames of a scene. The assumed situation is illustrated in Figure 1a. The cameras are working at the frame rate of 200 fps, capturing gray scale images of resolution 1024 × 1024. As briefly mentioned in the Introduction, this is mainly because an object appearing in the given space is assumed to be moving possibly with very fast speed. Image textures of such an object may be blurred out if long exposure is used. In addition, when using low frame rate, displacement between images of an object in two adjacent frames will be large, which makes it more difficult for an algorithm to find the object under tracking, because of larger possible searching space and greater appearance change.

In our setup, the two cameras are spaced not very close to each other, so that the 3D coordinates of any point calculated by triangulation [23] will have greater tolerance against small errors in point correspondences.

Images captured from cameras are processed in several stages, as coarsely illustrated in Figure 1b and Algorithm 1. Every time a new pair of frames is passed into the image processing system, we first detect a set of interest points from each of them. The result produced by a general purpose interest point detector is far from satisfactory for our purpose, and we apply an algorithm based on image gradient to detect interest points in this step. Detected interest points in both camera views, denoted by P0 and P1, are then passed into the matching process to calculate point correspondences for all object points maintained in the list of existing objects *O*. Well matched image points are then selected to estimate the motion parameters for every object and to update information for them. The relative pose change of an object allow the cameras to see more points than when that object is first observed. Thus, a method to link newly observed points in the scene with existing objects is proposed by using a dummy object, OD, to stock those points before testing their motion parameters against that of every existing object, $O_{i}\in O$. Well aligned points are then assigned to the corresponding object before otherwise being categorized as candidate points of a newly appearing unknown object. Whether this new object, which is temporarily labeled as OD, truly exists is verified taking account the number of total points consisting the object. If the number of points is larger than a threshold, which is decided concerning the assumed possible targets, the corresponding object will be added into the list existing objects. Finally, a new dummy object OD is generated from remained interest points P0 and P1, for the processing in the next time stamp. Each stage described here is detailed in the following subsections.

**Algorithm** **1:** Coarse processing flow for a single frame
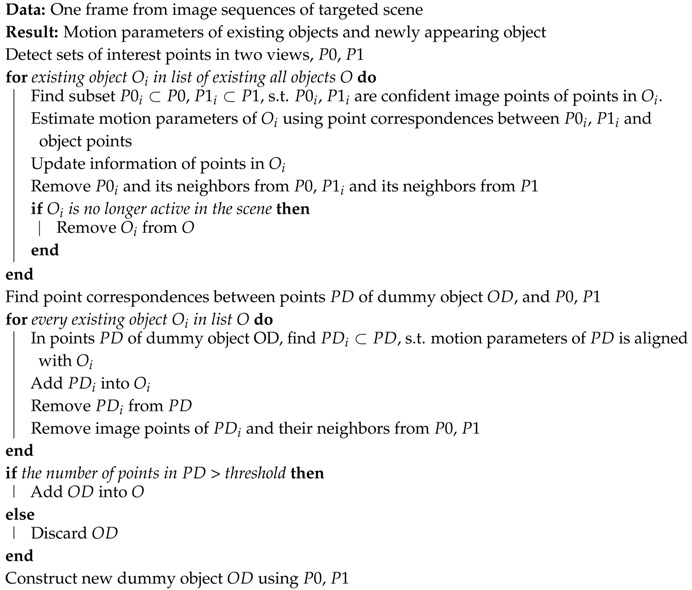


### 3.1. Detection of Interest Points

It is common to calculate a set of interest points among all pixels in a given image, before calculating local feature descriptors. Many alternatives exist for detecting interest points, but most of them try to find corner-like points [24], which is intuitive, because those points are more likely to be easily identified compared with points located at flat image area. This works in many situations when large number of potential corner-like points exist inside region of interest. However, on a fast moving object, points that are otherwise expected to be corner-like appear to be within flat area of an image because of motion blur. Consequently, only very few points, if any, can be detected using those detection methods. Robust estimation algorithms performs poorly if provided with only very small amount of good sample points, and is likely to fail in such cases.

**Figure 2:**
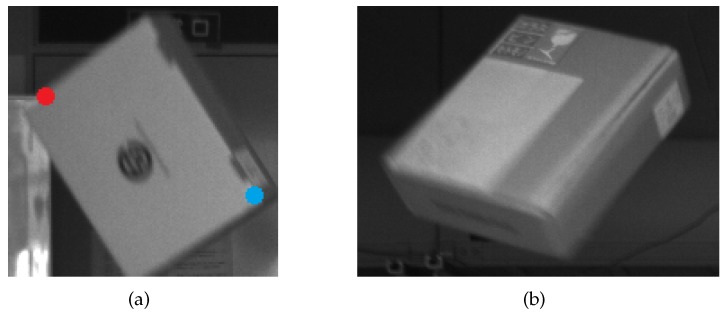
Example images illustrating motion blur in real scenes. (**a**) is a typical image patch of size 150 × 150 for a moving object cropped from an image frame of 1024 × 1024. The edges are not distinct compared with that in the background. The image of the trajectory one object point travels in unit exposure time covers several pixels, depending on its velocity and relative position with respect to the camera. In this example, the range around the red dot on the left side is about 10 unit pixels, and is about 3 pixels around the blue dot on the right side. The exact position of a point is difficult to be precisely calculated without concrete knowledge about the motion history of the target, which is not possibly known from one image frame. (**b**) This is a severe case caused by the short distance from camera.

When an object is moving, a physical point on the object generally maps to a curve segment in its image. This segment typically covers a range of several pixels in an image frame. Figure 2 shows real examples of motion blur cropped from raw image frames. It is difficult to say any of the points lying on the segment can better represent the corresponding object point than any other. Thus, there is a chance for any of them to be used as a candidate of an interest point.

Based on this consideration, in our method, we first detect a set of points as candidates of potential interest points using thresholded image gradient. The threshold needs to be set low to catch those blurred area where interest points may locate. The threshold at a point is set to be a percentage of the averaged intensities in the neighbor of the point. Then, the steps to calculate feature descriptors for image patches surrounding those points using algorithms such as [18,19,20,21,22] are followed, and then whether those points are appropriate for use as interest points is verified. We have tested some of these algorithms, and currently choose BRISK in our implementation. The actual algorithm chosen will affect the detection result, which is also discussed in Section 5.2.

Because the total number of such candidates is likely to be very large, calculating feature descriptors for all of them is inefficient from an aspect of computation cost. Since we are only interested in moving objects, it is natural to focus on the foreground and omit all points that are considered to be in the background. We use background subtraction for this purpose.

The verification of whether a candidate point can be used as a good interest point is via comparison of its feature descriptor against those of other candidate points lying inside its neighbor. This step is similar to the gradient based detection step in a general purpose interest point detector. The difference, however, is that our method searches interest points in their feature space, rather than in illuminance space.

Denoting f(p) the feature descriptor at a point *p*, and $p^{\prime }$ another candidate point in *p*’s neighbor $P_{neighbor}$, we select *p* as one interest point if for every $p\subset P_{neighbor}$, the following condition holds:

$$\frac{|f\left(p\right)-f\left(p^{\prime })\right|}{\left|p-p^{\prime }\right|}>threshold.$$

The threshold here is tuned heuristically, according to the local feature descriptor algorithm f(p).

**Figure 3:**
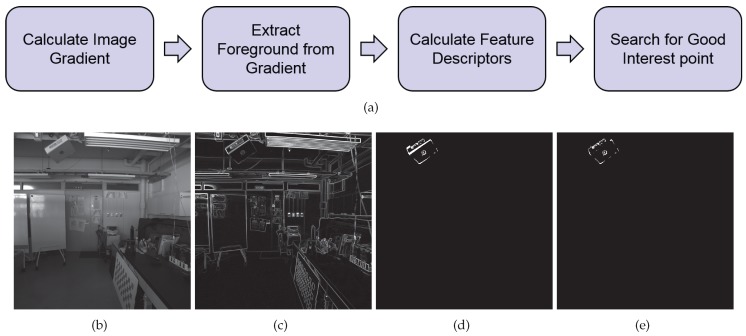
Coarse processing flow of interest points detection and its examples: (**a**) the main work flow to detect interest points from an image frame; and (**b**–**e**) examples of an input image, its image gradient, the map of candidate interest points, and the map of interest points after verification, respectively.

Figure 3 illustrates the main processing flow of interest points detection. Some processing steps of lower importance are abbreviated for conciseness.

As mentioned in the Introduction, general purpose local features do not distinguish between foreground pixels and background pixels, and may lead to undesired results, affecting subsequent processing. In our method, interest points are only possibly detected near edges (corners included) of foreground object, this greatly reduces the risk of incorrect matching when trying to find point correspondences. By searching within the feature space of candidate interest points, only points that can be locally well identified are selected. These points can be thought of corner-like points in the feature map of an image. They will be used for finding point correspondences in subsequent processing.

### 3.2. Point Correspondence along Time

Point correspondences between object points and image points are basically searched via matching of feature descriptors, using flann based matcher [25]. Assuming any point at a time cannot travel too far because of limited speed, images of that point in two adjacent image frames will, consequently, be close to each other. This constraint can largely reduce the total searching space of point correspondence for any existing object point, and thus speed up the detection.

### 3.3. Estimation of Pose and Position

The estimation of pose and position adopts the robust estimation routine RANSAC algorithm [16]. We assume objects appearing in the scene are rigid. Then, the location of any point on the object is fixed in its local coordinate system. Note that no information of any kind is assumed to be provided about the structure of objects under observation, so the actual coordinate of any point is not known to the system, and only relative position between points can be inferred from reconstructed point coordinates. We have to set a local coordinate system, under an assumption that a certain objects is about to have its origin located at the center of detected points. We also set the pose of an object to be an identity matrix when it is detected at the first time.

Here, we use a three-vector $P={\left(X,Y,Z\right)}^{T}$ to denote the 3D coordinates of a point on the object in its local coordinate system. The transformation parameters for a rigid body in 3D Euclidean space can be represented using a 3 × 3 matrix *R* for rotation, and a three-vector *t* for translation. Then any point *P* on the object will be transferred to one in the camera coordinate system by

(1) $$P^{\prime }=RP+t.$$

This point is then projected onto the image plane in camera at the point $p^{*}$, satisfying

(2) $$s\left[\begin{array}{c} p^{*}\\ 1 \end{array}\right]=AP^{\prime },$$

where $A=\left[\begin{array}{ccc} f_{x} & 0 & c_{x}\\ 0 & f_{y} & c_{y}\\ 0 & 0 & 1 \end{array}\right]$ is the camera intrinsics, and *s* the scaling factor. Note that the nonlinearity (or camera distortion) is assumed to have been corrected here, for conciseness.

We want to coarsely estimate *R* and *t*, in the sense that the re-projection error, between the image points $p_{i}^{*}$ calculated using estimated *R* and *t* and those $p_{i}$ directly observed in images, to be upper bounded for both cameras.

(3) $$error=\frac{1}{n}\sum_{i=1}^{n}\left|p_{i}-p_{i}^{*}\right|<threshold.$$

Here, *n* is the total number of points to be fitted in both cameras. The threshold is a constant number used in RANSAC algorithm, representing the tolerance of re-projection error in pixels. We set it to be 15, concerning the error range of motion blur.

**Figure 4:**
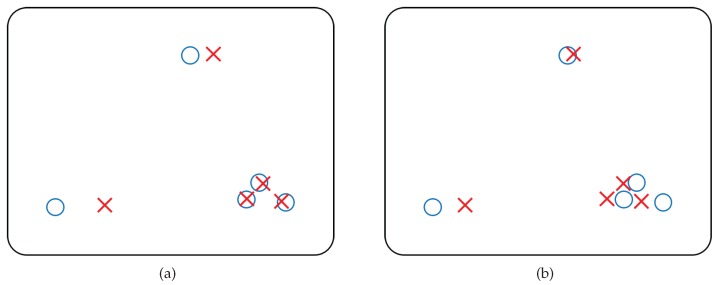
Estimation using non-uniformly distributed sparse point set. The circles in the block represent locations of image points observed directly, and the crosses that of estimated image points. The goal is to find a good transformation so that errors between circles and crosses are small. If every point is equally weighted, (**a**) may be a better estimation than (**b**) since the errors of the three points at the bottom right is much smaller, dominating the final error score. If the point in the left corner is labeled as an outlier by robust estimation algorithm, the final result will be a more extreme one. Although, from human intuition, (**b**) seems a more balanced transformation considering the distribution of sampled points.

This formula applies to situations where points are approximately uniformly distributed. It is commonly used for estimation with dense point set, where almost all image pixels are in use, so they lie on grids approximately equally spaced. However, when the distribution of sampled points are much different from a uniform one, as is the case in our approach, estimating using this cost function can lead to largely biased result. Figure 4 illustrates a simple example of this problem. Especially, when robust estimation algorithms are used, the problem may lead to a result that only points inside a cluster which contains large number of points but covers very small area in the image, is well fitted and labeled as inliers.

To avoid this problem, we introduce a weighting scheme for every image point, and use the weighted cost function in the robust estimation process. The cost function in (3) is modified to

(4) $$error=\sum_{i=1}^{n}w_{i}\left|p_{i}-p_{i}^{*}\right|<threshold.$$

The weight assigned to a point is decided as a function of its distance from other image points in the same view.

(5) $$w_{i}=\sum_{i=1}^{n}\sum_{j=1}^{n}{\left|p_{i}-p_{j}\right|}^{2}.$$

These weights are then normalized by the scaling factor ${\left(\sum_{i=1}^{n}w_{i}\right)}^{-1}$.

By introducing these weights, points located at sparsely sampled areas are given much higher importance, and thus can affect the cost function more compared with those at densely sampled area. This property greatly reduces the chance for the estimation process to poorly fit non-uniformly distributed image points and can serve as an effective solution for the stated problem.

After estimating the motion parameters, a simple Kalman filtering step [26,27] is applied. Some poorly estimated parameters may also be recognized and rejected in this step.

### 3.4. Updating Information for Object Points

Since local image feature descriptor is used as identifier of a point, and the appearance of any object point is changing along with the time, it is necessary to update the descriptor when possible. If point correspondences are perfectly taken for every object point, the updating procedure would be straightforward: just replace the old ones with the new. However, practically, this procedure is not simple. Some of the reasons are listed as below:

1. Not all object points can be matched to image points in every frame.
2. Because of motion blur, there is uncertainty within any detected image point, resulting imperfectly reconstructed coordinate in object coordinate system.
3. Affected by changes of background pixels in image patches surrounding a point, the feature descriptor attached to that point may vary rapidly, leading incorrect point correspondence across frames.

We need to find good point correspondences, and use them to direct how descriptor of a point shall be updated. The incorrectness of a point correspondence is evaluated using re-projection error calculated from estimated motion parameters.

Since the point correspondence between object point and image point is searched only inside neighbor of a limited range, re-projection error of this point must be upper bounded if the error is caused by incorrect matching. However, for cases caused by incorrect coordinate reconstruction, the error value typically has a much wider distribution parameterized by the error in its coordinate and motion parameters of the object. Thus, we distinguish between a bad point reconstruction and an incorrect point correspondence using a threshold on re-projection error. Points of bad reconstruction will be removed from an object, and points with bad matching will be taken equivalently as those that no good matching is found for. Then, all object points that are not matched to any image points are updated by re-calculating the descriptors for them after projecting those points onto images.

### 3.5. Adding Newly Detected Points into Existing Object

The detection of points is performed every time a new object appears in the scene. Since the whole structure of this object is not known at its first appearance, we have to associate points to an object when more points become visible in the scene.

Whether some points belong to a specific object is verified by comparing the motion parameters of those points and that object.

Since we assume that a moving object under observation is rigid, then, if a set of points belongs to a certain object, translation of every point in the set can be described using the motion parameters of that object. Use $R_{0}$ and $t_{0}$ to denote the pose and translation of an object at time $t_{0}$, respectively, $R_{1}$ and $t_{1}$ at time $t_{1}$. Then, the transformation between $t_{0}$ and $t_{1}$ must be the same for points in the same object, which means

$$dR=R_{1}R_{0}^{-1},$$

and

$$dt=t_{1}-dRt_{0},$$

apply to all points in the object.

It is not possible to uniquely calculate dR and dt for one point. Newly reconstructed scene points, which are temporarily stocked in the dummy object of Algorithm 1, generally contain points from multiple objects. Thus, we test those points in the dummy object against the $dR_{i}$ and $dt_{i}$ for every existing objects $O_{i}$. If the error is under a given threshold, the point is then considered aligned with the motion parameters of the corresponding object, and will be added into that object.

### 3.6. Generating New Object

Initially, no object is considered to be existing in observed space. An object is generated until some confident structure appears, so that motions of points consisting the object can be properly represented by a single set of motion parameters.

The generation of a new object is at the very late stage in the whole cycle of processing, as is shown in Algorithm 1. This is necessary because any structures that are aligned with existing elements are excluded from new structure so we can always use the fewest parameters to describe a given scene.

This idea is consistent with that of background subtraction. Background in a scene can be thought of as one large object, points of which are all constrained by a pair of static pose and translation. The difference is that this large object by default exists under this assumption, and has fixed parameters. Thus, subtracting background can be seen as a way to spare computational resources, and is basically equivalent to skipping the processing for this default object, since our interest is not on this object.

### 3.7. Point Correspondences across Views and Reconstruction of 3D Points

To reconstruct 3D points in space, we have to first find point correspondences between two cameras. There is not much prior information known about a targeted scene, except for the epipolar constraint available from camera calibration parameters, and an assumption that object points are continuous in space. Thus, for processing, we first compare the local feature descriptors of detected interest points and matched them across camera views. Then, we apply a filtering step in the displacement map calculated from point correspondences, rejecting jumping points and reducing noise. This is followed by a typical triangulation [23] step for reconstructing 3D coordinates for points. Reconstructed points are then stocked in the dummy object of Algorithm 1.

## 4. Experiment

### 4.1. Synthetic Scenes

**Figure 5:**
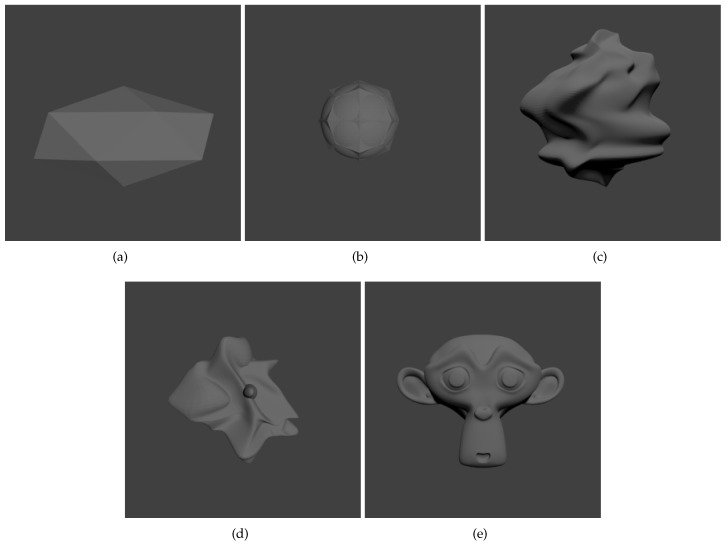
Objects used in synthetic scenes: (**a**) a low polygon mesh object; (**b**) a round shape object with shape corners; (**c**–**d**) two objects with smooth surface; (**e**) a monkey face, with rich features. Image sequences are rendered using either object from (**a**–**e**), under different conditions.

Here, we test our method using synthetic scenes and evaluate the results by comparing the estimated parameters with the true ones that are used to render the scenes. We use a set of five different objects, shown in Figure 5, as the target objects, rendering them separately under different conditions. We place each object in front of a textured background plane to approximate a cluttered environment. The target objects are moved in space using known trajectories and poses, which are saved along with the rendered images.

As will be discussed in Section 5.2, the automatically calculated origin for the local coordinate system of an object will lead to large biases in estimated positions of that object. Thus, the incorrectness of estimated positions cannot be well defined using the absolute errors between the estimated and the true positions. However, the estimated poses can be evaluated using the saved orientation of a target, because the poses are independent of any translation in space.

The pose errors are calculated using the angles between the true and estimated orientation differences over most recent 20 frames. If we denote the true rotation matrix from $t_{0}$ to $t_{1}$ as *R*, the estimated rotation as $R^{*}$, then the error matrix $R_{err}$ is defined to be the matrix which correct the estimated pose to the true one, satisfying

$$R_{err}R^{*}=R.$$

$R_{err}$ can then be calculated from *R* and $R^{*}$, before being converted to a rotation vector in axis/angle representation using Rodriguez’s formula. The norm of the vector is the error angle. This angle is divided by the true rotation angle calculated from *R*, and the error percentage is used as the error measure.

We temporarily exclude the filtering steps mentioned in Section 3.3, and use the estimated results directly from robust estimation process. We render, for every target object, five sequences of image pairs, each consisting of 500 frames. The results are listed in Table 1.

The conditions in Table 1 differ in their light sources, target motions, and background images. The error percentages in the table are averaged over frames in all consistent subsequences, i.e., in each subsequence, the target object is continuously recognized as the same object. Estimation fails sometimes, leading to the consequence that the target under tracking is lost for a time. A few frames are required before the target can be detected again. We list the number of frames summed over all consistent subsequences in a total of 500, in Table 2, for each scenario corresponding to those in Table 1. This number can not reach 500 because at least two frames, in our current setting, are required for an object to be detected and initialized.

We notice the differences in errors between columns in Table 1, and in the numbers of frames in Table 2. This means that the estimation precision actually varies according to the appearance of an object. This is because the estimation process is based on a sparse set of interest points which are identified with their local features. Object **(a)** is the simplest, low polygon mesh among all objects, with very little visual feature, and we see the errors with object **(a)** is basically worse than other objects. The tracking failure with **(a)** is also frequent, so the number in Table 2 is obviously smaller than any other columns. Objects **(c)** and **(d)** have more visual clues compared with **(a)**, but their surfaces are smooth and very few corner points exist, so the point correspondences may not be precisely calculated. The errors with them are larger than **(b)** and **(e)**, which have comparatively more points associated with distinct local features.

The overall error percentage on the synthetic scenes is about 10% or larger. Thus, when an object rotates $90^{{}^{\circ}}$, the error angle may exceed $10^{{}^{\circ}}$. The error may also be accumulated along time, as will be discussed in Section 5.2. The system can only coarsely estimate the motions, and can not be applied to applications that requires good precision.

### 4.2. Real Scenes

In this section, we demonstrate the experimental results tested in real scenes. However, first, we want to briefly introduce the setups for the experiments. The cameras used are spaced about half a meter away from each other, pointing at a space where moving objects are assumed to appear. Wide angle lenses are used for the digital cameras. Image distortion around the border of captured frames can not be completely corrected, so error trends to be larger around the border areas. Both cameras are directly controlled by the main PC, and they are synchronized when capturing. They work at the frame rate of 200 fps, sending 8-bit gray scale images in resolution 1024 × 1024.

Before conducting the experiments, we performed calibration for every single camera using Zhang’s method [28] implemented in OpenCV https://opencv.org/, and then applied stereo calibration subsequently.

It is important to evaluate the error range of the coordinate of a point reconstructed by triangulation. Because the triangulation is fully adopted in our system, the triangulation error should be viewed as one of the system errors, and all the results shown in the following sections shall be discussed with this error under concern.

The triangulation error are measured by placing a board with known pattern into the scene at random positions, and calculating distances between reconstructed pairs of points on the pattern. Errors estimated in this process are up to about 10% of the actual physical distances directly measured from the board.

Then, experiments on estimating motion parameters for unknown objects are conducted. Toys and paper boxes are used as the main targets in our experiment. No models of those objects is given as input to the system, and all information is collected from images while estimating the motion parameters of those targets.

Those objects are thrown into the scene by hand from outside in different directions, in a free manner. Light conditions are not strict, as long as the light sources are stable during short time interval, and is strong enough for the target objects to be observed. More textures on an object will help in the process of detecting interest points, so better lighting condition is desired, but is not always a requirement. We could see that some area in the image examples is very dark.

We randomly experiment on several such situations, calculate the motion parameters for them, and visualize those data so we can verify whether the system works as expected. Since it is difficult to get precise data as ground truth for the motion parameters of those tested objects, re-projection error is used as a measure of the incorrectness of reconstructed object structure and estimated motion parameters.

The following gives an example for when only one single moving object exists in the scene, detailed in Section 4.2.1, and another example for scene with multiple targets, shown in Section 4.2.2.

#### 4.2.1. Single Object

We tested our system against several real scenes for single object. One of them is selected and discussed here in this section. A white toy is used in this example as the target object. All of the figures in this section is created from the same scene. The total duration of the whole sequence is about 0.7 s, with more than one hundred frames captured.

**Figure 6:**
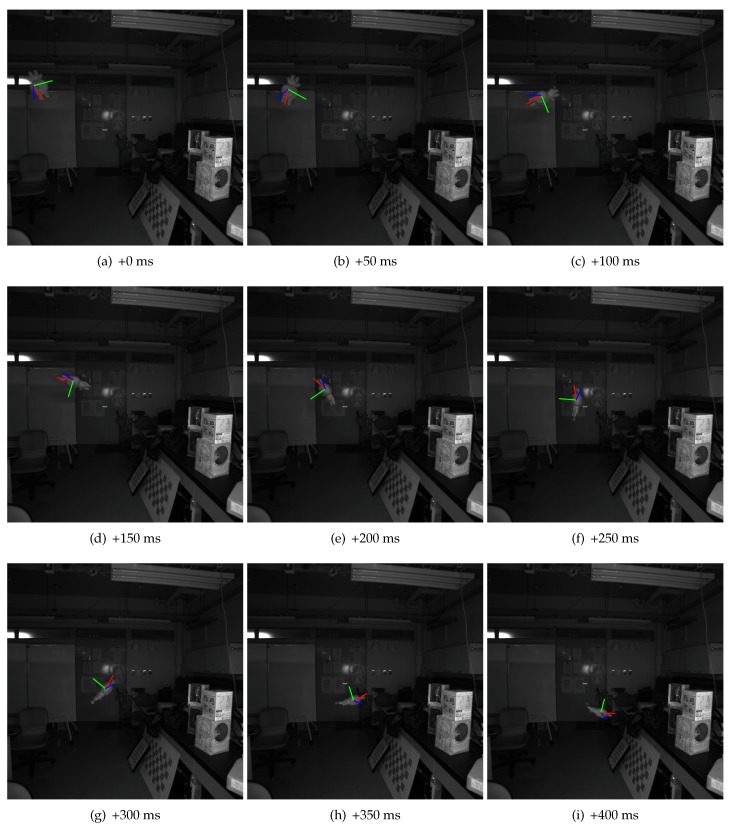
Estimated pose for single moving object. These images are selected every 10 frames from a sequence of totally more than 100 frames, from one of the cameras, labeled 0. The camera is configured to capture images at 200 fps, so the interval between any two frames shown here is 50 ms. The axes in these frames is calculated using the estimated pose and position of the target object, which is a white toy in this example. Since the object coordinate system is not predefined, and not possibly known by the system, only the changes in pose matter. We could see that all the estimated poses are as expected, and the positions of the axes are aligned with the toy.

A set of virtual axes are used to visualize estimated positions and poses for the detected object in the captured images. Figure 6 shows some of the frames collected from the sequence. Note that the object coordinate system used is generated by the algorithm, so, for poses, only the changes in pose matter in the system. We could see that all the estimated poses are as expected. The positions of the axes are also aligned with the toy. The size of axes actually depends on the estimated distance from the camera, although it is a little difficult to be noticed from this figure.

**Figure 7:**
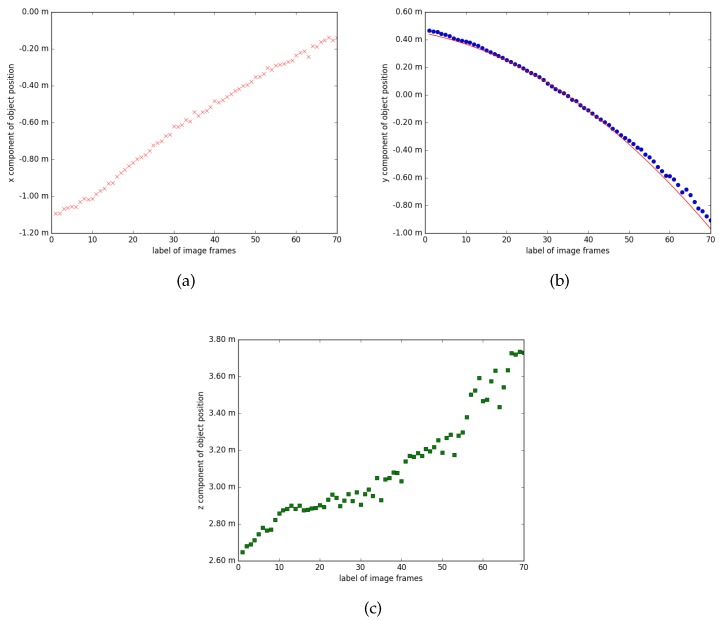
Estimated positions for single moving object. The dots in these figures show the estimated positions of the toy for the same scene in Figure 6. The data are outputs directly from the robust estimation routine, before any filter being applied. (**a**–**c**) are respectively the x, y, z component of the position, along time. Here, x axis is in the horizontal direction, y in the vertical direction, and z in the depth direction, in accordance with the coordinate system of one of the cameras, labeled 0. Since the forces on the target in free falling are dominated by gravity, we would expect the trajectory of the toy to be a parabola. As is shown in (**a**,**c**), the x and z components can indeed be fitted to straight lines, and in (**b**), the y component can be fitted with a parabola shown as the red curve.

Figure 7 plots the trajectory of estimated positions, represented with three components x, y, z in the coordinate system of one camera, labeled 0. These components correspond to the horizontal direction, the vertical direction, and the depth direction of an image, respectively. One would expect the x and z components to be a straight line, and y to be a parabola for the case because the target is thrown into the scene. As shown in these figures, the x and z components can indeed be fitted to straight lines, although the latter is relatively noisy, while the y component can be fitted with a parabola shown as the red curve in Figure 7b. Because the camera optical axis is unlikely to be strictly aligned with the earth horizon, it is possible that the x and z components may also have some curvature.

**Figure 8:**
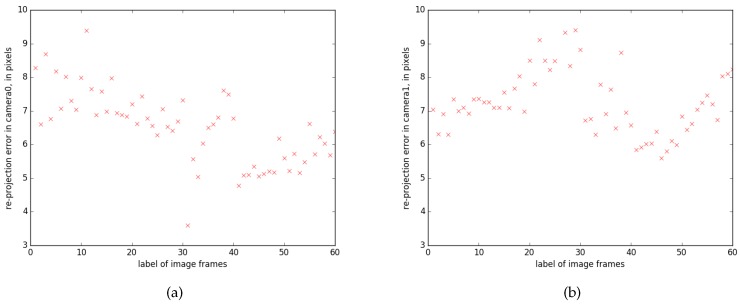
**Re-projection error in two camera views along time.** These images show the averaged errors over all points maintained in the detected object, in pixels. The errors are measured between re-projected object points using estimated parameters, and their matched interest points that are directly detected from images: (**a**) the errors measured in camera 0; and (**b**) the errors measured in camera 1. Although the errors range up to 10 pixels, this is considered to be reasonable, compared with the image resolution of 1024 × 1024 and the level of motion blur is typically 3–10 pixels.

Re-projection errors are measured between re-projected object points using estimated parameters, and their matched interest points, which are directly detected from images. Figure 8 shows the averaged errors over all points maintained in the detected object, for both cameras. Although the errors range up to 10 pixels, this is considered to be reasonable, compared with the high image resolution of 1024 × 1024 and the level of motion blur is typically 3–10 pixels.

**Figure 9:**
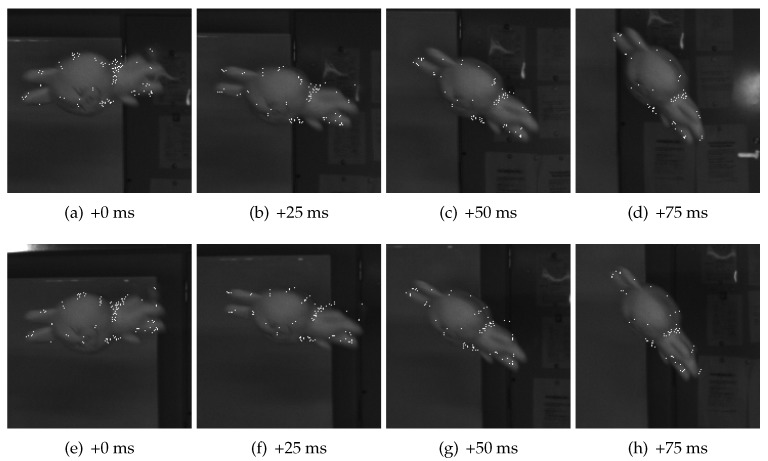
Object points projected onto images using estimated parameters. The four figures in the first row (**a**–**d**) are from one of the cameras labeled 0, and those at the bottom (**e**–**h**) are from the other labeled 1. Images of back projected object points are shown using white dots. In the images, we see that points in different frames and views are approximately located at the fixed positions with respect to the toy, as expected.

Figure 9 can be interpreted as a more intuitive way of visualizing the re-projection errors. In this figure, object points are projected back onto the images, represented by white dots. As we could see, most of the points are approximately located at their fixed positions on the object. Note that the total number of points is not a constant, because some of the points are removed from the object point set if they are labeled as badly reconstructed ones or not useful ones, while some new points are added if their motion parameters are aligned with the object.

We can see from these results that the moving object, which is a freely failing toy, is detected, and its motion parameters in subsequent frames are coarsely estimated. We consider this example to be successful. We have tested our system against many scenes in a similar way, the majority are successful, showing satisfactory detection and estimation results. However, we also find some failure cases. We discuss these cases in Section 5.

#### 4.2.2. Multiple Objects

The previous example is for the situation when only one object appears in the target space. However, the algorithm designed for our system also works for multiple objects, if no severe occlusion between objects occurres in the scene. This section shows an example where two objects co-exist in the scene.

**Figure 10:**
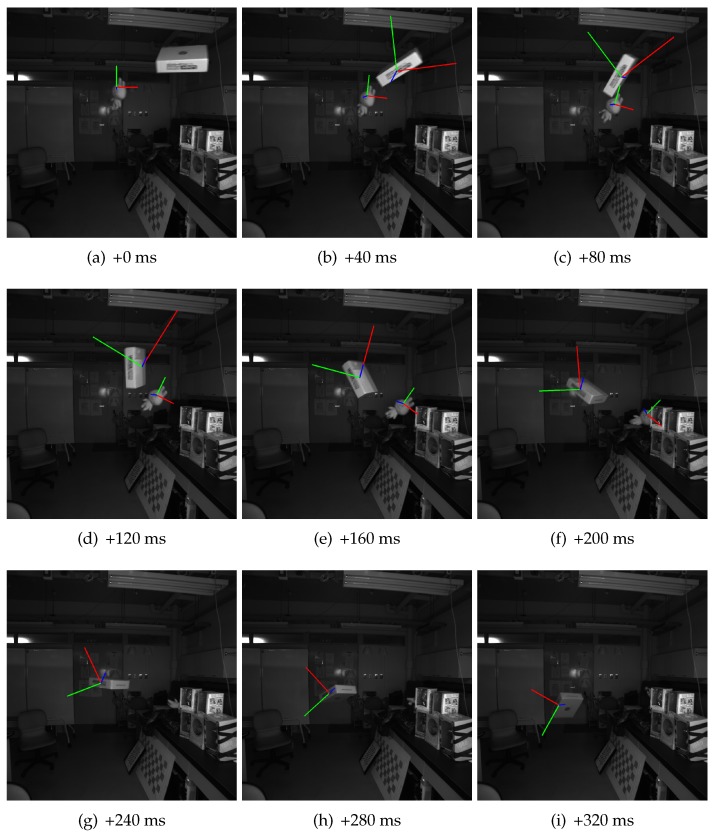
Estimated poses for multiple moving objects. This example uses the same setup as in Figure 6, changing the target objects to a toy and a paper box. Because it is difficult to throw the two objects at exactly the same time, the duration for the two to co-exist in the scene is shorter, so here we pick up images every eight frames, resulting in the 40 ms time interval between adjacent images shown here. Since the toy appears earlier than the paper box, at first in (**a**), only the toy is detected. Some frames later, as we see here in (**b**), the paper box is also detected and tracked. After (**f**), the toy is occluded by some other objects in the scene and is no longer visible, thus, from (**g**), only the paper box is present in the scene. We could see that all the estimated poses are as expected.

A toy and a paper box are used in this example. Similarly, they are thrown into the scene from outside in a free manner. Because it is difficult to keep more than one object flying in the space, frames from a shorter sequence are used, as shown in Figure 10. Our system successfully detected the two objects, and also estimated the poses and positions for them. We do not show the detailed data as we did in Section 4.2.1, since they are considered redundant and unnecessary.

However, we have to point out that the system can only coarsely estimate the poses of detected objects. The limitations are further discussed in Section 5.2 in detail. Here, we could see that the estimated poses are gradually drifting on the box. The height of the box is very short, and the area is small when viewed from its side. Thus, good object points are comparatively fewer, leading to larger estimation error. The flat shape and absence of textures also exacerbate the situation. This case is similar to the synthetic object **(a)** in Section 4.1.

## 5. Discussion

### 5.1. About Experiments

In Section 4.1, we generate synthetic scenarios using crafted objects, rendering long sequences, and saving their motions along with every rendered image pair. By comparing the estimation errors between different targets, we see that the error is influenced by the appearance of the target. Manually crafted low polygon mesh objects generally lack rich features, and estimation on such objects easily fails. The overall precision of estimation is not high, but the approximate motion can be calculated. We think the system can only coarsely estimate the motion, as stated in the tile of the paper.

In Section 4.2, we show results of two examples used in our experiments on real scenes. We can see from the single object example, that very little texture is available on the toy which is also indistinct, if we look at the cropped images in Figure 9. Under such conditions, general purpose feature detectors failed to find good points on moving objects, when they are tested against images from real scenarios. In addition, many of them are sensitive to light conditions and perform poorly when the scene is not sufficiently illuminated.

Images of target objects in our experiments typically take only very small portions in the whole captured frames. For the robust estimation algorithm to work properly, it is desired that the total number of input sample points is not small. This is because the possibility for the algorithm to fit within an incorrect subset of input samples will increase, since the algorithm is designed based on a statistical assumption. Using our proposed method for detecting points, we get tens to hundreds of points on a single object, which is a satisfactory amount.

The weighting scheme in Section 3.3 plays an importance role in the estimation step. In our system, not all observed points of a target are reconstructed. This is desirable from an aspect of computation cost. However, it leads to the fact that the object points project to sparsely non-uniformly distributed points in the image plane. Simply adopting the conventional error function which is used for dense point set causes problem, i.e., areas within which image points are densely distributed may be over fitted while other areas may be completely ignored. This will lead to incorrect estimation result because the absolute error level in point correspondence is high. By introducing the weighting scheme, the risk of poorly estimating positions and poses of an object is largely reduced.

Other techniques used in our system help to maintain the point set for every detected object.

All these proposed techniques contribute to the final performance of our system. Overall, we consider the system to be capable of detecting unknown moving objects and estimating the motion parameters for them.

### 5.2. Limitations

We have to point out that there are several limitations in our system, which cause some instability, and sometimes lead to undesired result.

The first one is related to the interest points detection and their matching. Our method for interest point detection makes use of image textures on target objects. As a general requirement in texture based methods, the texture at a physical point on target must be approximately identical when viewed from different perspectives. This requires the reflection on the material to be isotropic. If this condition is not satisfied, point correspondences may not be correct. Then, when not enough textures exist in images, the number of detected interest points may be too few to correctly estimate the motion parameters. A failure caused by this is unlikely to be recovered using a simple technique like filtering. The failures with object **(a)** in Section 4.1 are examples of this case. Besides, since we use local features to decide good interest points among candidates, the actual algorithms chosen for calculating feature descriptors can affect the final result of detection. We currently use BRISK [20] in our implementation. However, we are considering using more powerful tools such as learning based features in our future work. Works in [29] shows the possibility to use convolutional neural networks to calculate point correspondences.

The second is about the error in estimated poses. As discussed in previous sections, error exists in reconstructed points using triangulation method. This error cannot be easily modeled and compensated, and is considered as one kind of system error. In addition, the error in the matching process, the total error in reconstructed 3D coordinate is likely to have a large upper bound. Although some bad points are rejected in the robust estimation step, the precision of reconstructed object points is not high. Thus, we can only coarsely estimate the poses for objects. These errors may get accumulated during a long period, which is undesired. We notice the drift of estimated poses in Figure 10. This remains to be a task in the future.

The coordinate origin in object coordinate system may also cause problems. The object coordinate system of an object is automatically generated, once that object is first detected. Since no concrete information about the object structure is provided, the calculated origin may be biased, away from the actual weight center, as we can see in Figure 6 and Figure 10. Trajectory calculated under such a coordinate frame is consequently a composition of the actual object trajectory and a component that depends on the object pose and the error vector from the true origin to the assumed one. Since the estimated poses may not be precise, it is difficult to compensate this error. Whether the filtering step can effectively reduce this error remains a question. When the changes in poses are gradual and simple, estimated trajectory of an object will look similar to the true trajectory of rotation center. However, when the changes in poses are large and complex, the estimated trajectory will look strange, and the absolute error will also be large.

Finally, for multiple objects, occlusion will be a problem. Our system can handle the case if an object is only occluded in one of the cameras, because point correspondences in both views are used. However, currently, the system does not assume the case when an object is largely occluded in both cameras. When such occlusion happens, the system will behave as if an object disappears from scene.

## 6. Conclusions

We build a vision system to detect unknown fast moving objects within a given space, calculating their motion parameters represented by positions and poses. To achieve this goal, we first proposed a method to detect reliable interest points from images of moving objects, which can be hardly detected by general purpose interest point detectors because of motion blur. 3D coordinates reconstruction is performed using these points. A schedule is made updating points maintained for all detected objects, taking account for appearance and perspective changes. A weighting scheme is introduced to adapt the robust estimation procedure used for dense point set to the case for sparse set, reducing the potential risk of biased estimation. We conclude from the experiments that our system is capable of detecting multiple unknown moving objects and coarsely estimating their positions and poses. However, the precision of estimation is not high, and the error trends to accumulate over long period.

## Figures and Tables

**Table 1:** Error in estimated poses.

Object	(a)	(b)	(c)	(d)	(e)
condition 1	15.60%	9.22%	10.21%	14.58%	9.19%
condition 2	16.99%	9.96%	10.05%	14.11%	11.03%
condition 3	13.71%	10.84%	11.65%	14.15%	10.93%
condition 4	11.86%	10.99%	11.31%	12.62%	11.69%
condition 5	15.25%	11.94%	11.32%	11.61%	9.96%

**Table 2:** Number of frames summed over consistent subsequences.

Object	(a)	(b)	(c)	(d)	(e)
condition 1	471	498	498	498	498
condition 2	459	498	496	498	496
condition 3	443	498	495	498	496
condition 4	427	498	495	495	493
condition 5	403	496	496	496	495
